# Microbial reduction of Fe(III)-bearing clay minerals in the presence of humic acids

**DOI:** 10.1038/srep45354

**Published:** 2017-03-30

**Authors:** Guangfei Liu, Shuang Qiu, Baiqing Liu, Yiying Pu, Zhanming Gao, Jing Wang, Ruofei Jin, Jiti Zhou

**Affiliations:** 1 Key Laboratory of Industrial Ecology and Environmental Engineering, Ministry of Education, School of Environmental Science and Technology, Dalian University of Technology, Dalian, 116024, China; 2 Chemistry Analysis & Research Center, Faculty of Chemical, Environmental & Biological Science and Technology, Dalian University of Technology, Dalian, 116024, China

## Abstract

Both Fe(III)-bearing clay minerals and humic acids (HAs) are abundant in the soils and sediments. Previous studies have shown that bioreduction of structural Fe(III) in clay minerals could be accelerated by adding anthraquinone compound as a redox-active surrogate of HAs. However, a quinoid analogue could not reflect the adsorption and complexation properties of HA, and little is known about the effects of real HAs at environmental concentration on bioreduction of clay minerals. Here, it was shown that 10–200 mg l^−1^ of natural or artificially synthesized HAs could effectively stimulate the bioreduction rate and extent of Fe(III) in both iron-rich nontronite NAu-2 and iron-deficient montmorillonite SWy-2. After adsorption to NAu-2, electron-transfer activities of different HA fractions were compared. Additionally, Fe(II) complexation by HAs also contributed to improvement of clay-Fe(III) bioreduction. Spectrosopic and morphological analyses suggested that HA addition accelerated the transformation of NAu-2 to illite, silica and siderite after reductive dissolution.

Iron is the fourth most abundant element in the Earth’s crust. Iron-bearing clay minerals account for up to 50% iron in the soils and sediments^1^,^2^,^3^,^4^. The variation of the valence states of structural iron in clay minerals tends to affect many different aspects of their physicochemical properties (e. g., surface area, water swellability and cation and anion exchange capacity, etc.), which can further influence nutrient cycling, plant growth and contaminant migration in the environment^1^,^2^,^3^,^4^.

The reduction of Fe(III) in the lattice of clay minerals could be achieved either chemically with reductants like sulphide, dithionite, hydrazine and citrate, or microbially with growing or resting cells^1^,^2^,^3^. During the past two decades, bioreduction of clay minerals has been studied with a wide range of bacteria and archaea, some of which could utilize clay-Fe(III) as electron acceptor to support their growth^1^. A capacity for reduction of clay-Fe(III) has been found with dissimilatory iron-reducing bacteria^5^,^6^, sulfate-reducing bacterium^7^, methanogens^8^,^9^ and certain (hyper)thermophilic strains^10^. As the major components of natural organic matter in terrestrial and aqueous environments, humic substances (HS) are recognized as capable of stimulating bioreduction of Fe(III) minerals^11^.A previous study^6^ performed 20 years ago used HS at 2 g l^−1^ to reduce clay-Fe(III), but this concentration is significantly greater than natural concentrations (i.e., 0.1 to several hundred mg C per litre)^12^, thus not representing the likely environmental processes. Remaining studies on HS-stimulated bioreduction of clay-Fe(III) applied anthraquinone-2,6-disulfonate (AQDS) as a redox-active analogue/surrogate of HS^1^. However, besides redox active moieties, real HS also contain many metal ion complexing groups. The complexation of Fe(III)/Fe(II) by HS has been suggested to increase the bioavailability and reducibility of Fe(III) (oxyhyr)oxides minerals^11^,^13^,^14^. Moreover, the sorption behavior of HS on iron minerals, which may affect the capacity for electron transfer and the bioavailability of mineral surface, has been found to be very different from that of AQDS^13^,^14^. It might be inappropriate to use results obtained with AQDS to completely reveal impacts of HS on clay-Fe(III) reduction in nature. Additionally, the sorption of humic acids (HAs) by clay minerals could result in HA fractionation and formation of the clay-HA complexes, which are commonly found in natural environment^15^. To the best of our knowledge, the roles of different HA fractions in improving clay mineral bioreduction have not been elucidated.

Based on the knowledge gap outlined above, the main objectives of this study were: (1) to investigate the effects of different HAs at naturally relevant concentrations on microbial reduction of clay-Fe(III), (2) to elucidate the influences of adsorption and complexation activities of HA on microbial reduction of clay-Fe(III).

## Results

### Increased bioreduction of clay-Fe(III) by HA

**Figure 1:**
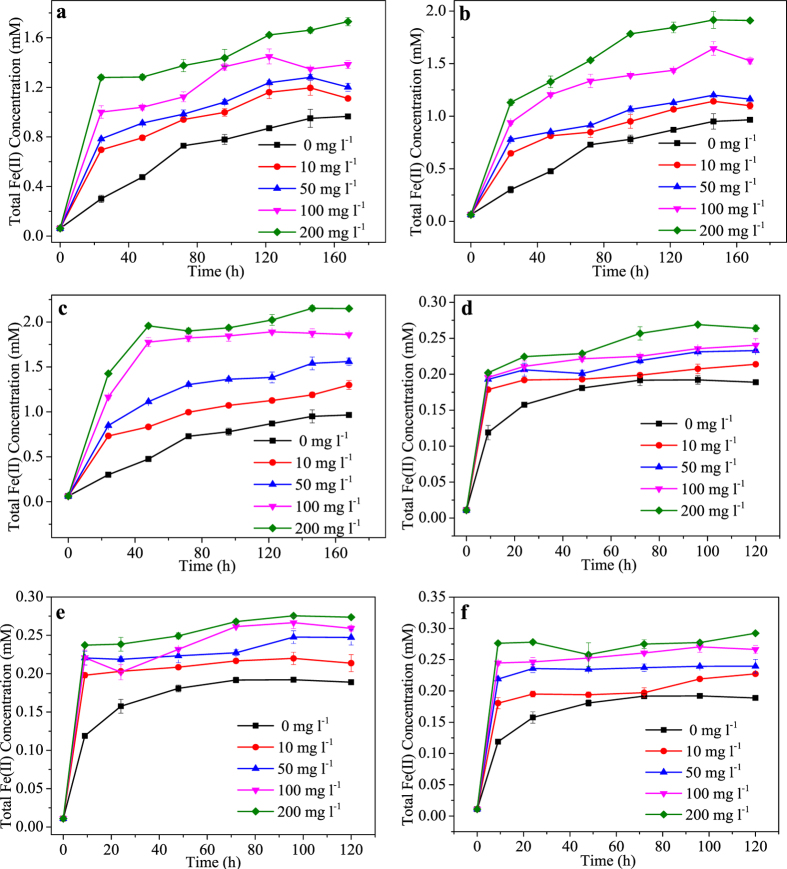
Increased bioreduction of clay minerals by HA. Bioreduction of NAu-2 (**a–c**) and SWy-2 (**d–f**) by MR-1 in the presence of 10, 50, 100 and 200 mg l^−1^ of (**a,d**) AHA, (**b,e**) SRHA and (**c,f**) ESHA. Error bars represented standard deviation (n = 3). Significant differences based on the one-way ANOVA (p < 0.05).

All the three tested HAs enhanced microbial reduction of Fe(III) in the two typical clay minerals (Fig. 1 and see Supplementary Fig. S1). For NAu-2 bioreduction, total Fe(II) concentration increased steadily to around 0.8 ± 0.0 mM in 170 h in the absence of HA. With the addition of HA, NAu-2 was reduced rapidly in the initial 24 or 48 h, which was followed by a slower reduction phase and gradually leveling-off Fe(II) concentration. Both the extent and initial rate of Fe(III) reduction increased with the increase of HA concentration (10–200 mg l^−1^). In the absence of HA, around 22% Fe(III) in NAu-2 was reduced by MR-1 in 170 h with an initial Fe(II) production rate of 10 μmol l^−1^ h^−1^. In the presence of Elliott soil humic acid (ESHA), which showed the best stimulation capacity among the three tested HAs, the reduction extents were generally 8.1 ± 0.3%-27.1 ± 0.1%, 4.7 ± 0.1%-9.4 ± 0.2% and 4.9 ± 0.2%-10.4 ± 0.5% higher than those observed in systems containing no HA, or the same concentration of Suwannee river humic acid (SRHA) and Aldrich humic acid (AHA), respectively. The addition of 10 and 50 mg l^−1^ of different HA species resulted in similar increases in the initial Fe(II) production rate (2.5–3.2 times higher than that of control system without HA). However, when HA concentration was raised to 100 and 200 mg l^−1^, initial Fe(II) production rates (24 h) of systems containing ESHA were generally 12.0 ± 0.2%-24.4 ± 0.5% and 24.4 ± 0.7%-25.7 ± 0.1% higher than those obtained with SRHA- and AHA-supplemented systems, respectively.

For the bioreduction of SWy-2 which contains less Fe(III), elevated Fe(II) production by HA addition was also observed, although the stimulating effects were less significant. The improvement in Fe(II) production mainly occurred in the initial 12 h. Around 6.1 ± 0.1%-29.0 ± 0.6% and 1.1–3.4-fold increases in the extent and initial rate of Fe(III) reduction were achieved respectively by addition of different HAs. ESHA still demonstrated better stimulation capacity than the other two HAs. With the increase of HA concentration, Fe(II) production increased whereas the variation of initial reduction rate was very limited.

**Figure 2:**
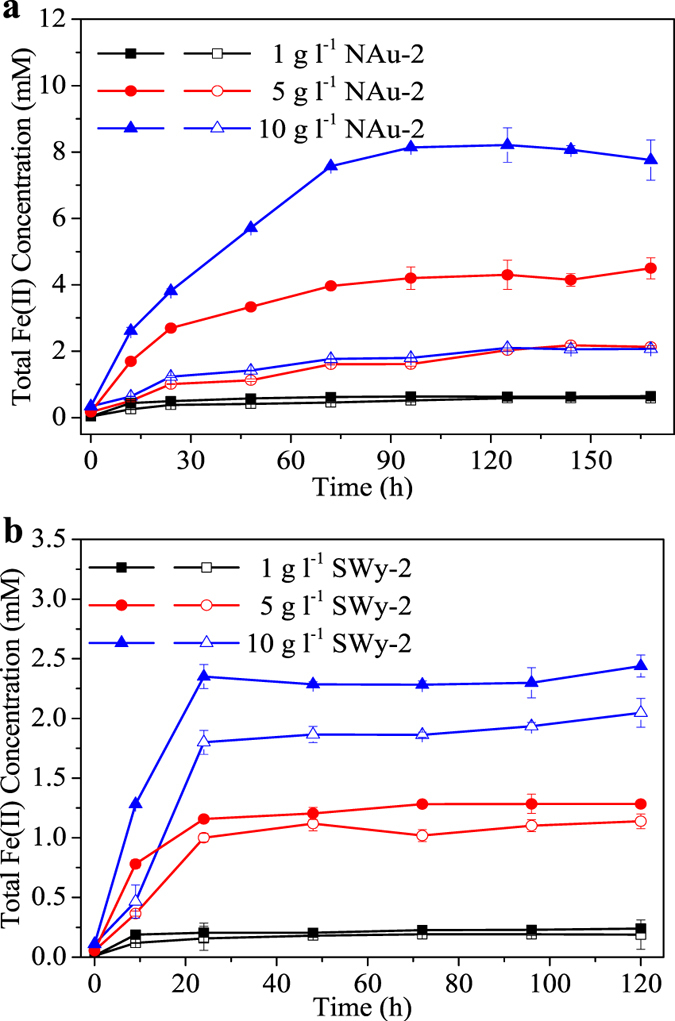
Increased bioreduction of different concentrations of clay minerals by HA. Bioreduction of 1, 5 and 10 g l^−1^ of (**a**) NAu-2 and (**b**) SWy-2 by MR-1 in the presence of ESHA. Solid and open symbols represent for systems with and without ESHA, respectively. Error bars represented standard deviation (n = 3). Significant differences based on the one-way ANOVA (p < 0.05).

Effects of ESHA on bioreduction of different concentrations of clay minerals were also studied (Fig. 2). In the absence of ESHA, Fe(II) generation increased significantly with the increase of initial SWy-2 concentration, whereas no significant difference could be found with Fe(II) production in systems containing 5 and 10 g l^−1^ NAu-2. In the presence of 50 mg l^−1^ ESHA, greater enhancements in Fe(III) reduction were found in systems containing higher clay mineral concentrations. Specifically, increases of 0.98, 2.37 and 5.69 mM in Fe(II) production were observed in systems containing 1, 5 and 10 g l^−1^ NAu-2, respectively. Increases of 0.05, 0.15 and 0.39 mM in Fe(II) production were obtained with systems containing 1, 5 and 10 g l^−1^ SWy-2, respectively.

### Sorptive fractionation of ESHA and impacts of different fractions on NAu-2 bioreduction

Pre-adsorption of ESHA to NAu-2 was carried out to separate different HA fractions before the start of microbial reduction. The adsorption process at room temperature could be described with a Langmuir isotherm model with a maximum adsorption capacity of 11.4 mg g^−1^ (see Supplementary Fig. S2). After adsorption, the polarity index (defined as atomic ratio of (N + O)/C) of unbound ESHA increased, which indicated the preferential sorption of low and non-polarity fractions by NAu-2 (see Supplementary Table S1). The ^13^C nuclear magnetic resonance spectroscopy (NMR) analysis suggested that aliphatic fractions were preferentially adsorbed by NAu-2 while aromatic ones were more likely to be left in solution (see Supplementary Fig. S3 and Table S2). This was corroborated by the decreasing ratio of ESHA absorbance at 465 and 665 nm (E_4_/E_6_) from 11.0 to 8.9 after adsorption.

**Figure 3:**
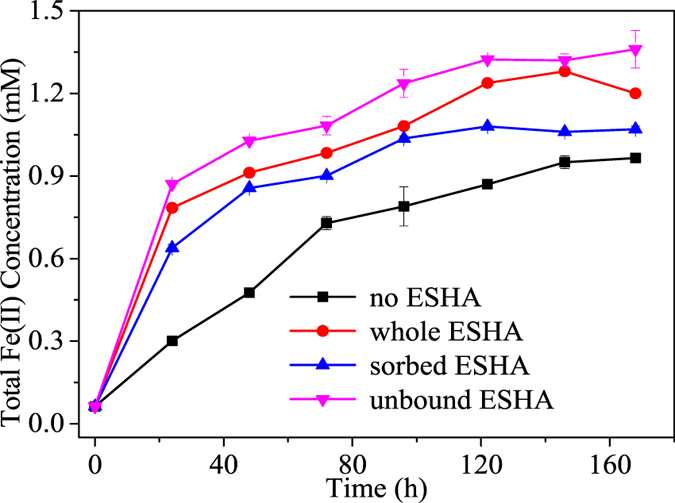
Increased bioreduction of NAu-2 by different fractions of ESHA. Whole ESHA and its sorbed and unbound fractions separated after adsorption to NAu-2 were compared for stimulating bioreduction of Fe(III) in NAu-2. Error bars represented standard deviation (n = 3). Significant differences based on the one-way ANOVA (p < 0.05).

**Figure 4:**
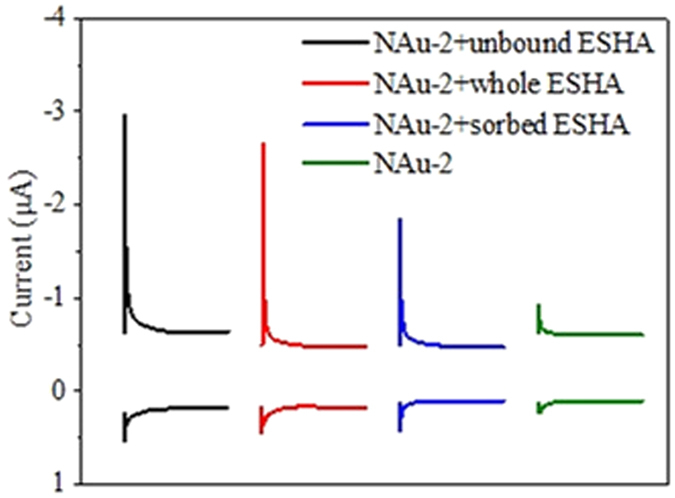
Reductive and oxidative current responses of NAu-2 and combinations of NAu-2 with different ESHA fractions. Mediated electrochemical reduction peaks were uppermost, with mediated oxidation peaks below.

As shown in Fig. 3, the presence of unbound or sorbed ESHA could both stimulate NAu-2 bioreduction. Surprisingly, the unbound fraction demonstrated even higher stimulating capacity than whole ESHA. As shown in Fig. 4 and Supplementary Table S3, NAu-2 alone had very limited electrochemical activities. Thus the electron accepting capacity and electron donating capacity (EAC and EDC) values of combinations were mainly contributed by ESHA components. All the different combinations of NAu-2 and ESHA demonstrated much higher values of EAC than those of their EDC, indicating the oxidized state of most redox active groups in these samples. The EAC value of suspension containing NAu-2 and unbound ESHA was 1.3 times higher than that of NAu-2 and whole ESHA mixture. And the EAC value of ESHA-sorbed NAu-2 was only 11.2% lower than that of NAu-2 and whole ESHA mixture. These results again indicated that both sorbed and unbound fractions of ESHA were redox-active and could stimulate electron transfer from cell to NAu-2.

### Influence of ESHA complexation with Fe(II) on NAu-2 bioreduction

It has been suggested that HA could improve reduction of Fe(III) (oxyhydr)oxides via forming complexation with Fe(II)^14^,^15^. The adsorption isotherm of Fe^2+^ to NAu-2 could be described with a Langmuir isotherm model (see Supplementary Fig. S4). In the presence of ESHA, the maximum adsorption capacity of NAu-2 for Fe^2+^ decreased from 0.83 to 0.75 mmol g^−1^.

Although the addition of ESHA greatly stimulated total Fe(II) production during microbial reduction of NAu-2, the increase of structural and adsorbed Fe(II) production (calculated from the difference of total and dissolved Fe(II)) was very limited. On the contrary, the percentage of dissolved Fe(II) in total Fe(II) increased from 4.6% to 17.2% (see Supplementary Fig. S5). An increase of about 0.20 mM in dissolved Fe(II) production was observed in the presence of ESHA. This is comparable to the theoretical maximum Fe^2+^ complexation capacity of ESHA (0.34 mM, its calculation based on Henderson-Hasselbalch equation and parameters provided by International Humic Substance Society, see Supplementary Text S1).

**Figure 5:**
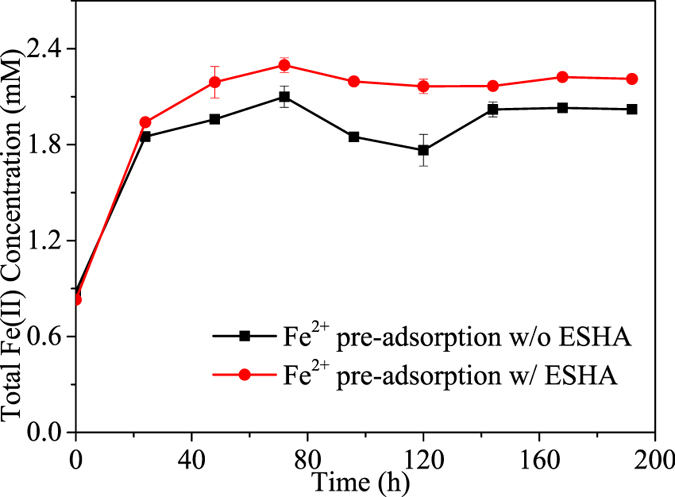
Effects of Fe^2+^ pre-adsorption on bioreduction of NAu-2. The reduction process was initiated after Fe^2+^ adsorption equilibrium was reached with or without ESHA. Error bars represented standard deviation (n = 3). Significant differences based on the one-way ANOVA (p < 0.05).

To further reveal the effects of ESHA complexation, microbial reduction of NAu-2 was carried out after reaching adsorption equilibrium of Fe^2+^ by NAu-2 in the presence or absence of ESHA. As shown in Fig. 5, when ESHA was present during pre-adsorption of Fe^2+^ by NAu-2, around 33.4% reduction was achieved in 192 h. However, only 27.3% NAu-2 reduction occurred when pre-adsorption was conducted without ESHA.

### Bioreduction products of NAu-2 in the presence of ESHA

Fe(II) production during NAu-2 bioreduction was accompanied by the release of other nontronite-associated elements, such as Al, Si, Mg and Ca (see Supplementary Table S4), indicating dissolution and structural damage of the clay minerals during reduction. It should be noted that the released Al and Si amounts in the ESHA-added system were significantly higher, confirming the promoted reduction and dissolution of NAu-2.

**Figure 6:**
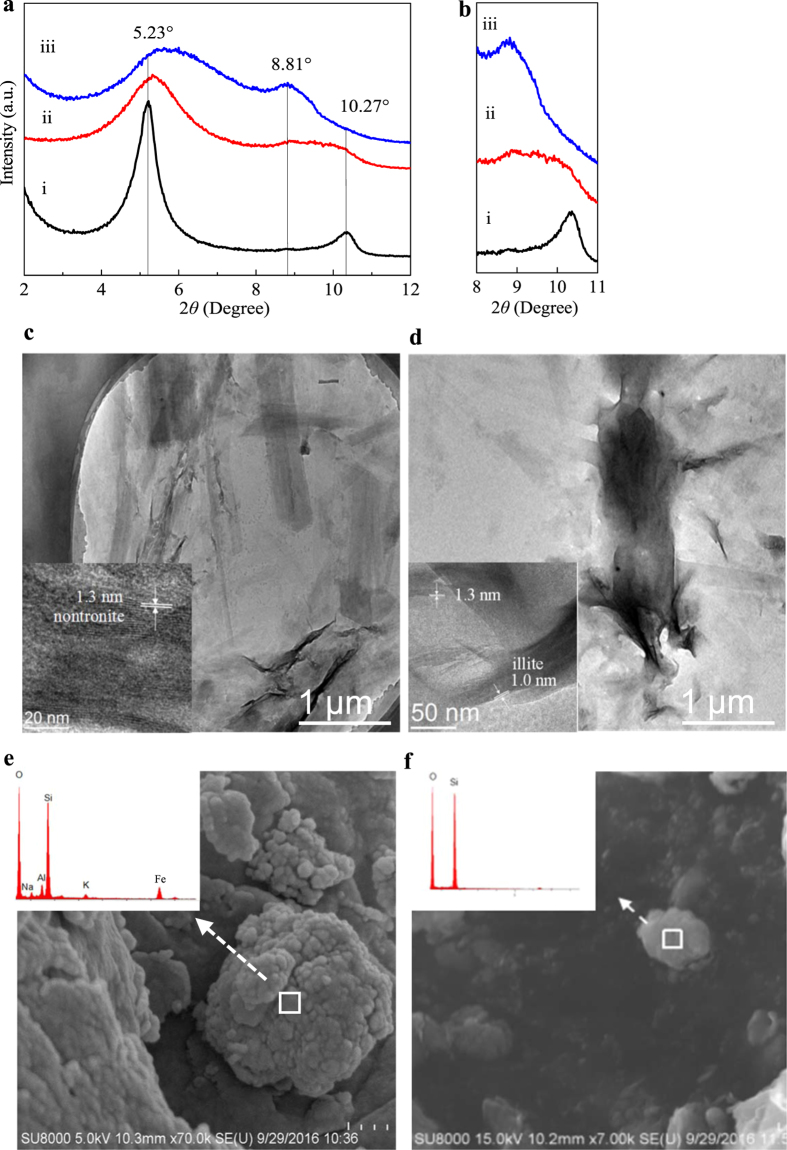
Characterization of NAu-2 and its reduction products. (**a**) XRD patterns of ethylene glycolated (i) NAu-2, and bioreduced NAu-2 (ii) without or (iii) with ESHA, (**b**) Detailed view of the peaks in the 2*θ* range of 8–11°, (**c,d**) TEM and HRTEM (inset) images of NAu-2 before and after bioreduction in the presence of ESHA, (**e,f**) SEM and EDX analyses of illite aggregation and spherical silica particle.

Mineralogical changes of clay minerals after bioreduction were detected with X-ray diffraction (XRD) spectrascopy (Fig. 6a,b). For (001) peak of NAu-2 (2*θ = *5.23°), decreased intensity, broadened width and shifted position to higher 2*θ* were shown in the result after bioreduction. Correspondingly, the d(001) spacing shifted from 16.9 Å to 16.4 Å (without ESHA) and 15.3 Å (with ESHA). The (002) peak of NAu-2 (2*θ* = 10.27°) also broadened or even disappeared after bioreduction. Moreover, a new peak corresponding to (001) peak of illite was identified at 2*θ* = 8.81° after reduction. It should be noted that the presence of ESHA generally resulted in greater extents of changes in the XRD pattern. Similar changes of XRD pattern were also observed after SWy-2 bioreduction (see Supplementary Fig. S6).

Transmission electronic microscopy (TEM) observation found that most lath-shaped NAu-2 particles were degraded and associated closely with MR-1 cells (Fig. 6d) after bioreduction. Energy dispersive X-ray (EDX) analysis connected to scanning electronic microscopy (SEM) identified the formation of illite (with high Al/Si ratio and K content) and silica after reduction (Fig. 6e,f). High resolution TEM (HRTEM) analysis found irregular particles with a layer spacing of 1.3 nm in unreduced NAu-2 samples. After bioreduction, the finding of 1.0-nm layer spacings revealed the formation of illite^3^ (Fig. 6d). Time-course HRTEM observation performed at 18, 48 and 72 h (see Supplementary Figs S7–S9) indicated earlier appearance of illite structure and higher illitization contents in the presence of ESHA. For instance, after 18 h of reduction, no sign of illite formation was observed in the absence of ESHA, whereas layer spacings of illite (1.0 nm) were identified at the edges of NAu-2 matrix (with layer spacings of 1.3 nm) in systems containing ESHA (see Supplementary Fig. S7d). In 48 h, although the layer spacings of most mineral particles remained at 1.3 nm, illite began to form at the edges of some particles in systems without ESHA. For systems containing ESHA, the layer spacings characteristic of illite were found at both edges and inner parts of mineral particles (see Supplementary Fig. S8f). In 72 h, although illite packets could be easily observed in samples from both systems, greater illitization extent was found in the presence of ESHA (ca. 15 vs. 5 layers) (see Supplementary Fig. S9b,f). A discrete illite phase was only observed after 72 h in ESHA-added systems (see Supplementary Fig. S10). Additionally, some irregular particles observed in the products of both ESHA-added systems in 18 h and control system without ESHA in 48 h were believed to be siderite, based on their 0.36-nm lattice fringes^16^.

## Discussion

HS ubiquitously exist in natural environments and have been well known to be capable of stimulating bioreduction of iron (oxyhydr)oxide minerals^13^. However, except for a much earlier study utilizing HA at an unreasonably high concentration^6^, almost all currently available studies on mediated reduction of Fe(III)-bearing clay minerals used AQDS instead of real HA. And little was known about the effects of HS on microbial reduction of Fe(III)-bearing clay minerals. For the first time, it was demonstrated that bioreduction of clay-Fe(III) could be enhanced by HA at meaningful environmental concentrations. As with the results from mediated γ-FeOOH bioreduction^17^, the terrestrial ESHA with higher aromaticity showed better stimulating capacity than both aqueous SRHA and artificially prepared AHA.

Even in the presence of AQDS, the complete bioreduction of clay-Fe(III) is generally unachievable due to factors such as system energetics limitation, the adsorption of released Fe(II), and accumulation of solid phase product^1^. Although the variation of microbial species, and diverse NAu-2 concentrations and incubation periods used in different studies made it difficult to directly compare the capacities for stimulation of electron transfer, the extents of reduction obtained here were generally comparable to those reported previously using AQDS, cystine and cysteine as electron shuttling compounds (see Supplementary Table S5). However, the initial reduction rate in the presence of HA was obviously much lower than those obtained by adding AQDS (for bioreduction of 5.0 g l^−1^ NAu-2, 105.2 μmol l^−1^ h^−1^ (50 mg l^−1^ ESHA) in this study vs. 350.0 μmol l^−1^ h^−1^ (1 mM AQDS in ref. 9). Thus the reductive transformation of clay minerals might proceed with much slower kinetics in real environments.

Results of sorptive fractionation of natural organic matter on minerals have been disputed^18^,^19^,^20^. Preferred sorption of different fractions (e. g. polar vs. nonpolar, aromatic vs. aliphatic) on mineral surfaces might be caused by the use of different HAs and minerals. Here, less polar and aliphatic fractions of ESHA were preferentially adsorbed by NAu-2. Electrochemical and microbial reduction experiments identified redox and reduction-stimulating activities with both sorbed and unbound fractions. Considering the great adsorption differences between HAs and quinones on minerals^13^,^14^, the non-negligible contribution of sorbed HA fraction in stimulating clay-Fe(III) reduction could not be revealed when AQDS was used as a surrogate. The electrochemical redox activity of unbound ESHA and NAu-2 mixture was significantly higher than that of the NAu-2 and whole ESHA combination, corresponding to the further Fe(III) bioreduction observed in the former system. On the other hand, the higher aggregation of NAu-2 particles in the presence of whole ESHA might inhibit bioreduction by decreasing the exposure of mineral surfaces^14^.

The inhibition of clay-Fe(III) bioreduction by biogenic and clay-sorbed Fe(II) has been found in the presence of AQDS^21^. Besides stimulating electron transfer, ESHA could also improve microbial reduction of NAu-2 through Fe(II) complexation, which prevented the blockage of mineral surface sites and provided thermodynamically favorable conditions for Fe(III) reduction^15^. This again suggested that AQDS, having only redox activity, could not completely model the role played by HA in promoting clay-Fe(III) reduction.

Two different mechanisms have been proposed for microbial reduction of Fe(III) in different clay minerals: dissolution-precipitation and solid-state reduction^1^. It has been assumed that electron transfer compounds improved electron transfer from directions both parallel and perpendicular to the basal plane of smectite structure, thus enhancing the reduction and dissolution extent of smectite^1^. Release of compositional elements and detection of illite, siderite and silica as reduction products during ESHA-mediated bioreduction of NAu-2 was in support of the dissolution-precipitation mechanism.

Transformation of smectite to illite (S-I reaction) has long been recognized as an important reaction in clay mineralogy and geochemistry^1^. Abiotic S-I reaction typically requires conditions of 250–350 °C, 50–100 MPa and 4–5 months^22^. Kim *et al*. firstly found that reduction and dissolution of nontronite by *Shewanella oneidensis* MR-1 could lead to illite precipitation from aqueous solution^23^. Subsequently, the capacity for promoting of illitization has also been observed with other iron-reducing bacteria, sulfate-reducing bacteria and even methanogens^3^,^8^,^16^,^24^. The microbially driven S-I reaction is normally completed under ambient conditions in several days to two weeks^1^,^11^. Zhang *et al*. found that cysteine intercalated in the interlayer of NAu-2 facilitated illitization^25^. Later, Liu *et al*. indicated that dissolved cystine or cysteine could also enhance the reduction and illitization of smectite by *Shewanella* species^26^. Our findings suggested that the ESHA-enhanced clay-Fe(III) bioreduction also caused more rapid and higher extents of illitization. The illite structure always initiated at the edges and then developed into the inner regions of clay mineral particles, resulting in mixed layers of illite-smectite phases. Therefore, the contribution of HA in S-I reaction in nature cannot be neglected.

In summary, we demonstrated that after sorptive fractionation, HA at environmental concentrations could enhance reduction and illitization of Fe(III)-bearing clay minerals via electron transfer enhancement and Fe(II) complexation. Redox-active AQDS used in previous clay mineral reduction studies might not completely reveal the effects of real HA. Whether HS could also effectively enhance reductive transformation of Fe(III)-bearing clay minerals by methanogens, sulfate-reducing bacteria and even Archea strains, deserve further investigations.

## Methods

### Minerals, chemicals and bacterial strain

Two model clay minerals, i. e. nontronite NAu-2 (M^+^_0.72_-[Si_7.55_Al_0.16_Fe_0.29_][Al_0.34_Fe_3.54_Mg_0.05_]O_20_(OH)_4_, where M represents the interlayer cation (e. g., Ca, Na or K)) and montmorillonite SWy-2 ((Ca_0.16_Na_0.24_)-[Si_6.73_Al_1.27_][Al_1.45_Fe^2+^_0.01_Fe^3+^_0.12_Mg_0.44_]O_20_(OH)_4_) were purchased from the Source Clays Repository of the Clay Minerals Society (West Lafayette, IN)^4^. It has been shown that each gram of NAu-2 contains 4.1 mmol of Fe, of which 99.4% is Fe(III), and each gram of SWy-2 contains 0.4 mmol of Fe, of which 97.3% is Fe(III)^27^. AHA was purchased from Sigma-Aldrich. SRHA and ESHA were both obtained from the International Humic Substance Society.

Bulk NAu-2 and SWy-2 were thoroughly ground and soaked in 0.01 M NaCl solution for 24 h. Then the 0.5–2.0 μm size fraction was isolated by centrifugation (7600 *g*, 20 min). The obtained fraction was repeatedly washed with distilled deionized water (Milli-Q) until no Cl^-^ could be detected with AgNO_3_ test. Finally, the processed clay minerals were freeze-dried, ground and stored at room temperature.

HA sample was dissolved into ultrapure water (pH 7.0) and stirred for 4 h. The resultant brown liquor was filtered through 0.45 μm acetate cellulose membranes, purged with N_2_ for 20 min and then kept under anaerobic conditions in the dark. The dissolved organic carbon concentration of HA stock solution was determined with a total organic carbon (TOC) analyzer (Shimadzu TOC 5000 A, Japan).

Ethylene glycol and the other chemicals were all of analytical grade, obtained from Sigma-Aldrich, TCI or Sinopharm and used without further purification.

*S. oneidensis* MR-1 obtained from American Type Culture Collection (ATCC 700550) was routinely cultured in tryptic soy broth at 30 °C overnight. Then cells were harvested by centrifugation (10,000 *g*, 5 min), washed with and resuspended in 10 mM sterile piperazine-*N,N′*-bis(2-ethanesulfonic acid) (PIPES) buffer solution, and held anaerobically before use in the following reduction studies.

### Microbial reduction of clay minerals in the presence of HA

The experimental systems were 50-ml serum bottles containing 30 ml deoxygenated sterile 10 mM PIPES buffer solution, 0.03 g of NAu-2 or SWy-2, 3 mg of KCl and 10–200 mg l^−1^ of AHA, SRHA or ESHA, respectively. After inoculation of MR-1 cells (~1 × 10^8^ cells/ml), serum bottles were sealed with butyl rubber stopper, capped with aluminum cap, and placed into orbital shaker at 30 °C in the dark. In addition, bioreduction of different concentrations of NAu-2 (1, 5 and 10 g l^−1^) was also investigated in the presence of 50 mg l^−1^ ESHA.

Pre-adsorption was carried out before reduction experiments to reveal the effects of different HA fractions on clay-Fe(III) bioreduction. ESHA solution (50 mg l^−1^) and NAu-2 suspension (1.0 g l^−1^) were mixed and then gently shaken on orbital shakers for 24 h. After that, centrifugation (7600 *g*, 20 min) was conducted to separate unbound ESHA fraction remained in supernatant from sorbed ESHA fraction in the pellets. Then, 1.0 g l^−1^ NAu-2 was re-supplemented into the separated supernatant containing unbound ESHA, whereas equal volume of media solution was added to resuspend remained pellets of NAu-2 with sorbed ESHA. MR-1 cells were then innoculated to initiate reduction. Normal reduction experiments without pre-adsorption, and NAu-2 bioreduction without adding ESHA were also conducted in parallel as a control.

To study the contribution of HA-Fe(II) complexation on promoting microbial reduction of clay minerals, FeCl_2_ (0–0.6 mM) was added alone or together with ESHA (50 mg l^−1^) into NAu-2 suspension (1.0 g l^−1^) in serum bottles, which were then gently shaken on orbital shakers until reaching Fe^2+^ adsorption equilibrium. An equal concentration of ESHA was placed into bottles containing no ESHA to guarantee the same compositions of all reduction systems. And MR-1 cells were inoculated to start NAu-2 reduction.

Samples were periodically removed with sterile needles and syringes and analyzed for total and dissolved Fe(II) concentration in an anaerobic chamber.

### EAC/EDC measurement

Mediated electrochemical reduction and oxidation^28^ (MER/MEO) were performed to assay the redox activity of combinations of NAu-2 with different ESHA fractions (whole, sorbed and unbound ESHA). In a glass electrochemical cell closed with Teflon cover, glass carbon electrode, Ag/AgCl electrode and platinum plate electrode served as working, reference and counter electrode, respectively. The counter electrode and the working electrode were separated by a cation exchange membrane. The system was filled with 0.1 M phosphate buffer (80 ml, pH 7.0) containing 0.1 M KCl. Chronoamperometry measurements were conducted with an electrochemical workstation instrument (Chenhua CHI660D). When the electrode reached the desired potential (*E*_h_ = −0.49 V for MER and + 0.61 V for MEO), 1 mL of diquat dibromide monohydrate stock solutions (10 mM) for MER or 2 mL of 2,2′-azino-bis(3-ethylbenzothiazoline-6-sulfonic acid) diammonium salt stock solutions (4.6 mM) for MEO were spiked, respectively. Until re-attainment of constant background currents, 0.5 ml of suspensions containing 1.0 g l^−1^ NAu-2 and 50 mg l^−1^ of whole ESHA or corresponding sorbed/unbound fractions were added into the cell, separately. Then the reductive and oxidative current peaks of different combinations were obtained. All solutions were purged with nitrogen gas (99.9%) for 2 h before use. A reaction time of 2500 s was used to guarantee completion of reaction. The resulting current peaks were integrated to obtain the EAC and EDC values.

### Characterization and analysis methods

The dissolved and total Fe(II) concentrations were measured with phenanthroline assay^27^.

For elemental analysis of solution, filtered samples (0.2 μm) were mixed with HNO_3_ before being analyzed with an inductively coupled plasma mass spectrometer (Perkin Elmer Optima 2000DV). Control and reduced clay mineral samples were analyzed to identify the mineralogical and morphological changes after bioreduction. XRD was measured with a Scintag X1 diffractometer using CuKα radiation. TEM images of samples were taken with a JEOL JEM-2100 LaB6 TEM at 200 kV. SEM and EDX analyses were carried out with NOVA NanoSEM 450 operating at 5.0 or 15.0 kV.

Structural carbon distributions of whole ESHA and unbound ESHA after clay mineral adsorption were characterized with ^13^C nuclear magnetic resonance spectroscopy.

All experiments were performed at least three times. Differences were compared by a one-way analysis of variance (ANOVA) and *p*-value of <0.05 was considered significant. The data were analyzed using SPPS 19.0.

## Additional Information

**How to cite this article:** Liu, G. *et al*. Microbial reduction of Fe(III)-bearing clay minerals in the presence of humic acids. *Sci. Rep.* **7**, 45354; doi: 10.1038/srep45354 (2017).

**Publisher's note:** Springer Nature remains neutral with regard to jurisdictional claims in published maps and institutional affiliations.

## Supplementary Material

Supplementary Information
