# Supplementary material for: Microbial reduction of Fe(III)-bearing clay minerals in the presence of humic acids
Source: Sci Rep. 2017 Mar 30;7:45354. doi: 10.1038/srep45354 (PMC5371790; doi:10.1038/srep45354)
Supplement: Supplementary Information [file srep45354-s1.pdf]

Microbial reduction of Fe(III)-bearing clay minerals in the presence of humic acids

Guangfei Liu[1,*], Shuang Qiu[1], Baiqing Liu[1], Yiying Pu[1], Zhanming Gao[2], Jing Wang[1], Ruofei Jin[1], Jiti Zhou[1]

[1] Key Laboratory of Industrial Ecology and Environmental Engineering, Ministry of Education, School of Environmental Science and Technology, Dalian University of Technology, Dalian, 116024, China

[2] Chemistry Analysis & Research Center, Faculty of Chemical, Environmental & Biological Science and Technology, Dalian University of Technology, Dalian, 116024, China

*corresponding author:

Guangfei Liu, guangfeiliu@dlut.edu.cn

Text S1 Fe(II) complexation by IHSS Elliott soil humic acid (ESHA) at pH 7.0

The data and equation used for the calculation are available on the homepage of the International Humic Substance Society (IHSS, http://www.ihss.gatech.edu). For further information about the determination of acidic functional groups in HA, titration modelling, and the potential for iron complexation by HA see Ritchie and Perdue[1].

1.1 Modified Henderson-Hasselbalch equation[1]:

$$Q_{tot} = \left(\frac{Q_1}{1 + \left(K_1 [\mathrm{H}^+] \right)^{1/n_1}} \right) + \left(\frac{Q_2}{1 + \left(K_2 [\mathrm{H}^+] \right)^{1/n_2}} \right)$$

Table Parameters given for ESHA by IHSS

C content	Q_1	log K_1	n_1	Q_2	log K_2	n_2
58.13%	8.90	4.36	3.16	0.85	9.80	1.00

1.2 Calculation of the overall charge density for ESHA at pH 7:

$$Q_{tot} = \left(\frac{8.90}{1 + \left(10^{4.36} \cdot [10^{-7}] \right)^{1/3.16}} \right) + \left(\frac{0.85}{1 + \left(10^{9.80} [10^{-7}] \right)^{1/1.00}} \right)$$

$$Q_{tot} = \frac{8.90}{1.15} + \frac{0.85}{631.95} = 7.77 \, {}^{mequ}\!/_{gC}$$

1.3 Complexation capacity of ESHA for Fe(II) in cultures:

One mole acidity binds 0.5 moles of Fe^{2+}:

$C_{Fe(II)c} = Q_{tot}/2 = 3.88$ mmol Fe(II)/g C

Complexation capacity of ESHA :

$C_{Fe(II)HA} = C_{Fe(II)c}/0.5684 = 6.83$ mmol Fe(II)/g HA

Amount of Fe(II) per liter, complexed by dissolved HA in cultures :

$C_{Fe(II)} = C_{HAdiss} \times C_{Fe(II)HA}$

Calculation of Fe(II) complexing capacity of 50 mg/l ESHA is 0.34 mM Fe(II).

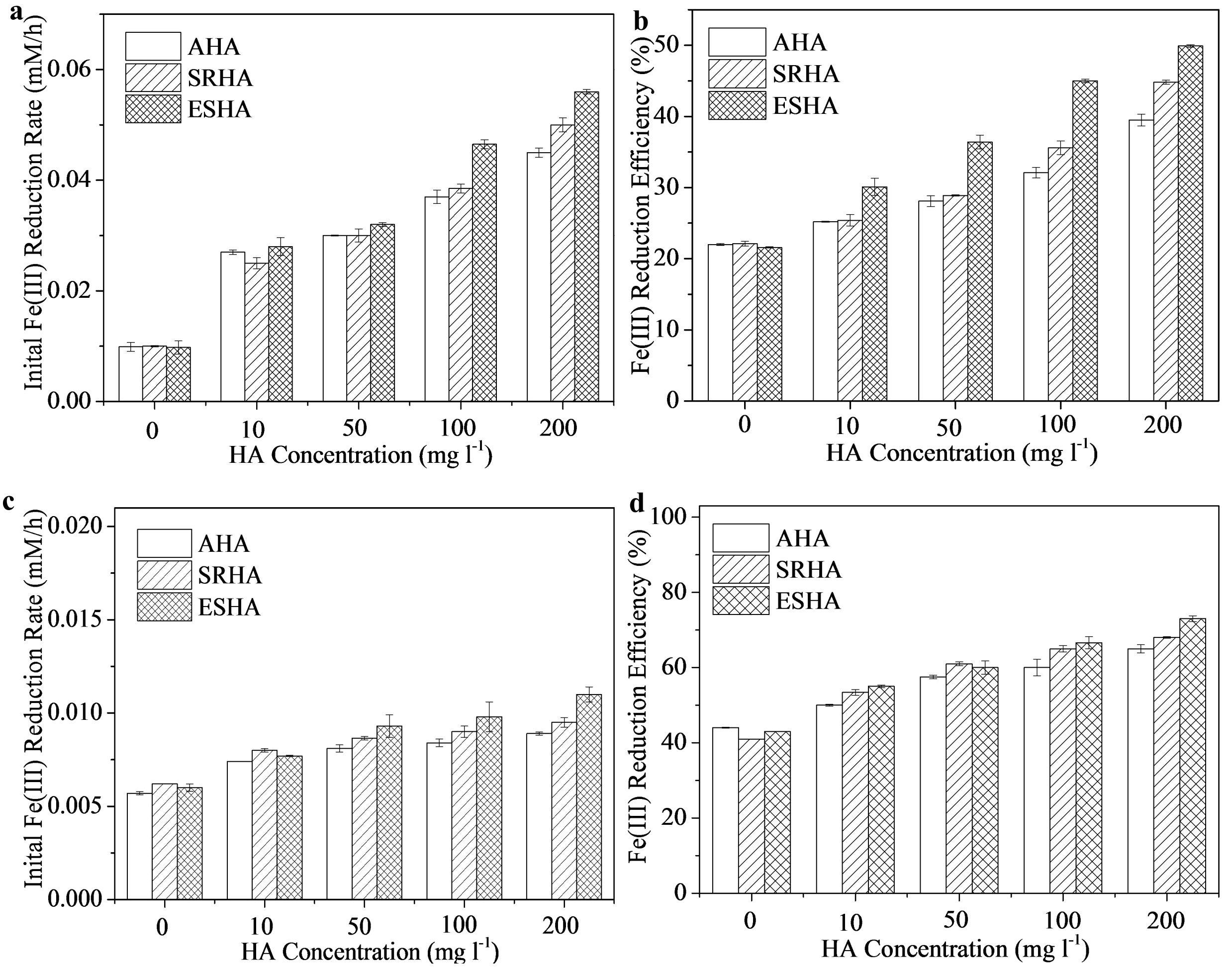

Fig. S1 Effects of different HAs on initial (a, c) reduction rate and (b, d) reduction content of Fe(III) in (a, b) NAu-2 and (c, d) SWy-2.

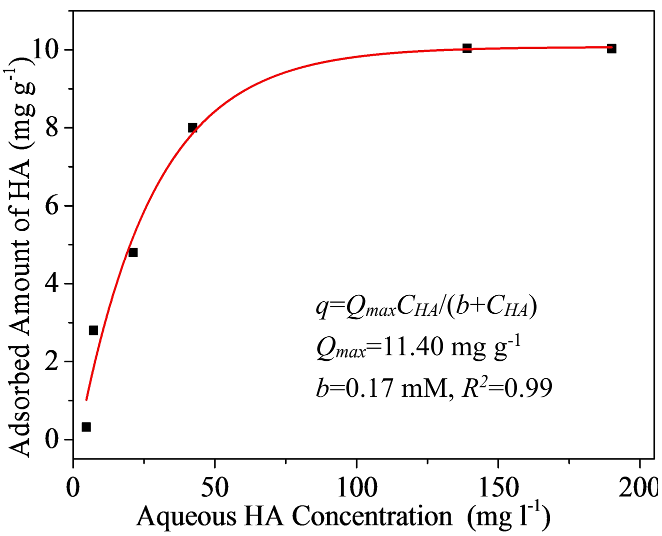

Fig. S2 Sorption of ESHA on NAu-2 at room temperature modeled by the Langmuir isotherm.

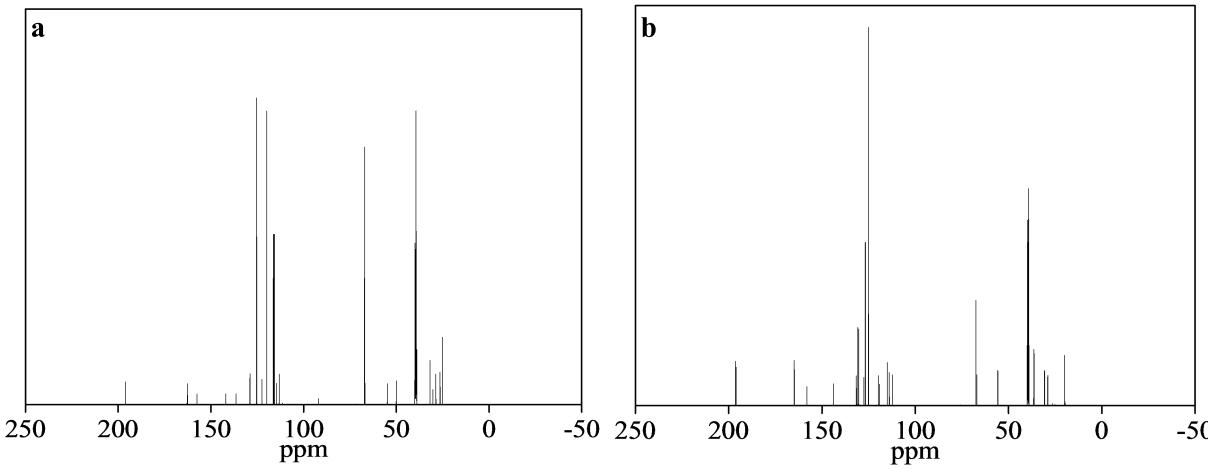

Fig. S3 ^{13}C NMR spectra for (a) whole ESHA and (b) unbound ESHA after adsorption.

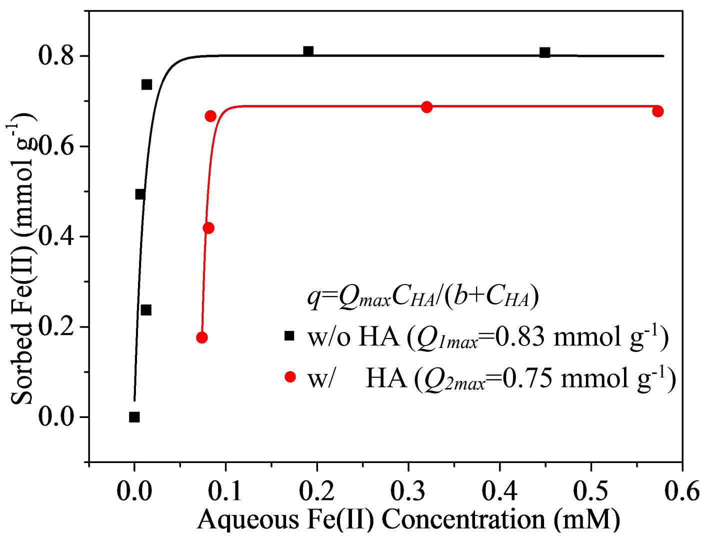

Fig. S4 Adsorption of Fe^{2+} to NAu-2 in the absence or presence of ESHA modeled with the Langmuir isotherm at room temperature.

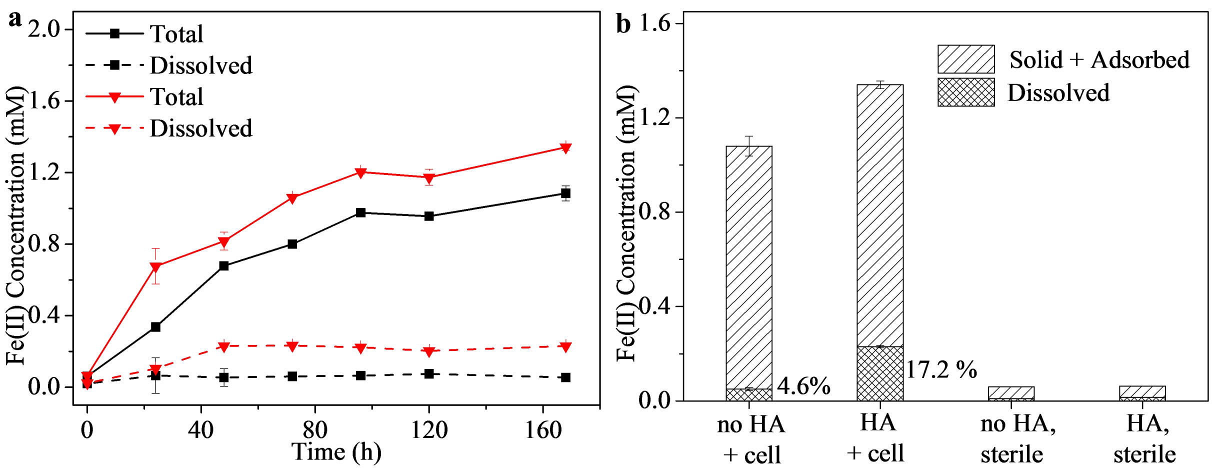

Fig. S5 Effects of ESHA on enhancing production of dissolved Fe(II) during NAu-2 bioreduction. (a) Measurement of total (solid line) and dissolved Fe(II) (dashed line) production during microbial reduction of NAu-2 in the presence (red triangle) or absence (black square) of ESHA. (b) Contribution of dissolved Fe(II) in total Fe(II) production of different systems.

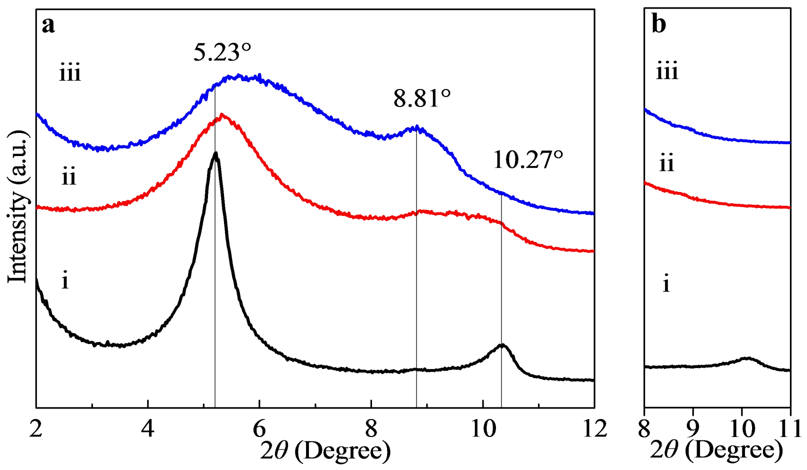

Fig. S6 (a) XRD analysis of ethylene glycolated (i) SWy-2, and bioreduced SWy-2 (ii) without or (iii) with ESHA. (b) Detailed view of the peaks in the 2θ range of 8-11°.

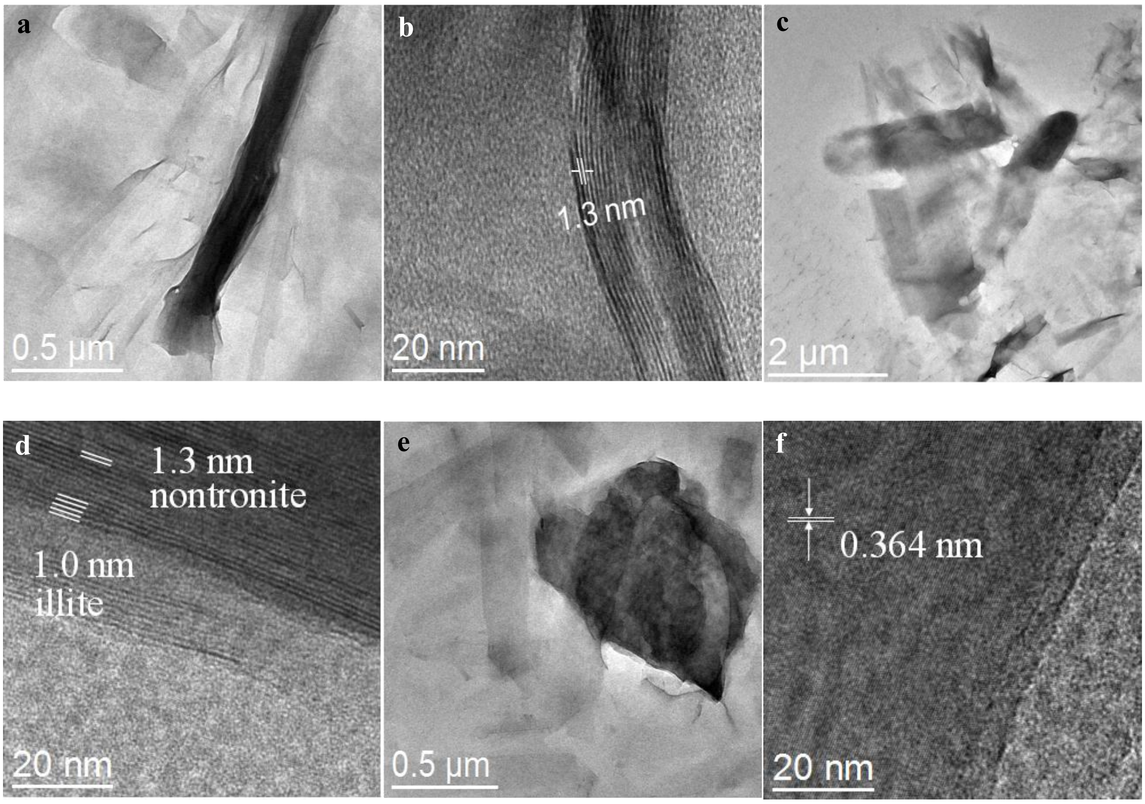

Fig. S7 TEM and HRTEM images of NAu-2 after 18 h of bioreduction. (a, b) Bioreduction of NAu-2 in the absence of ESHA, (c-f) Bioreduction of NAu-2 in the presence of ESHA, (e, f) Fe-rich precipitates with layer spacings typical of siderite.

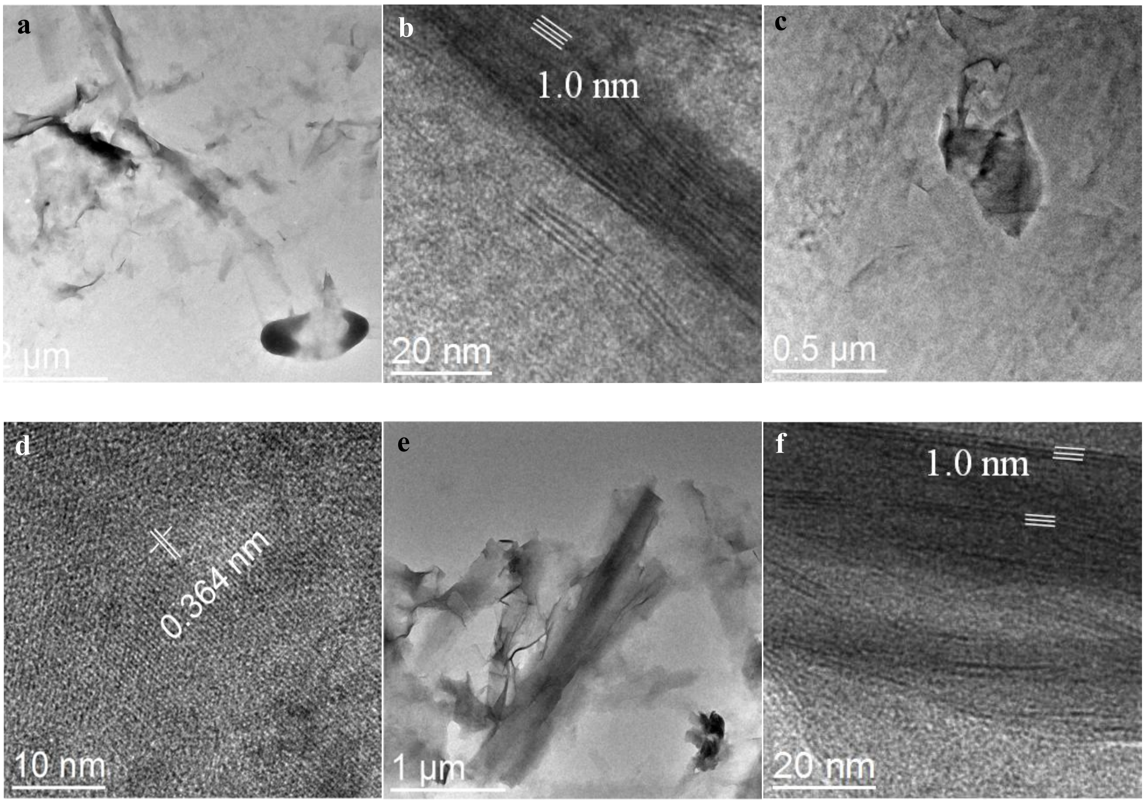

Fig. S8 TEM and HRTEM images of NAu-2 after 48 h of bioreduction. (a-d) Bioreduction of NAu-2 in the absence of ESHA, (c, d) Fe-rich precipitates with layer spacings typical of siderite, (e, f) Bioreduction of NAu-2 in the presence of ESHA.

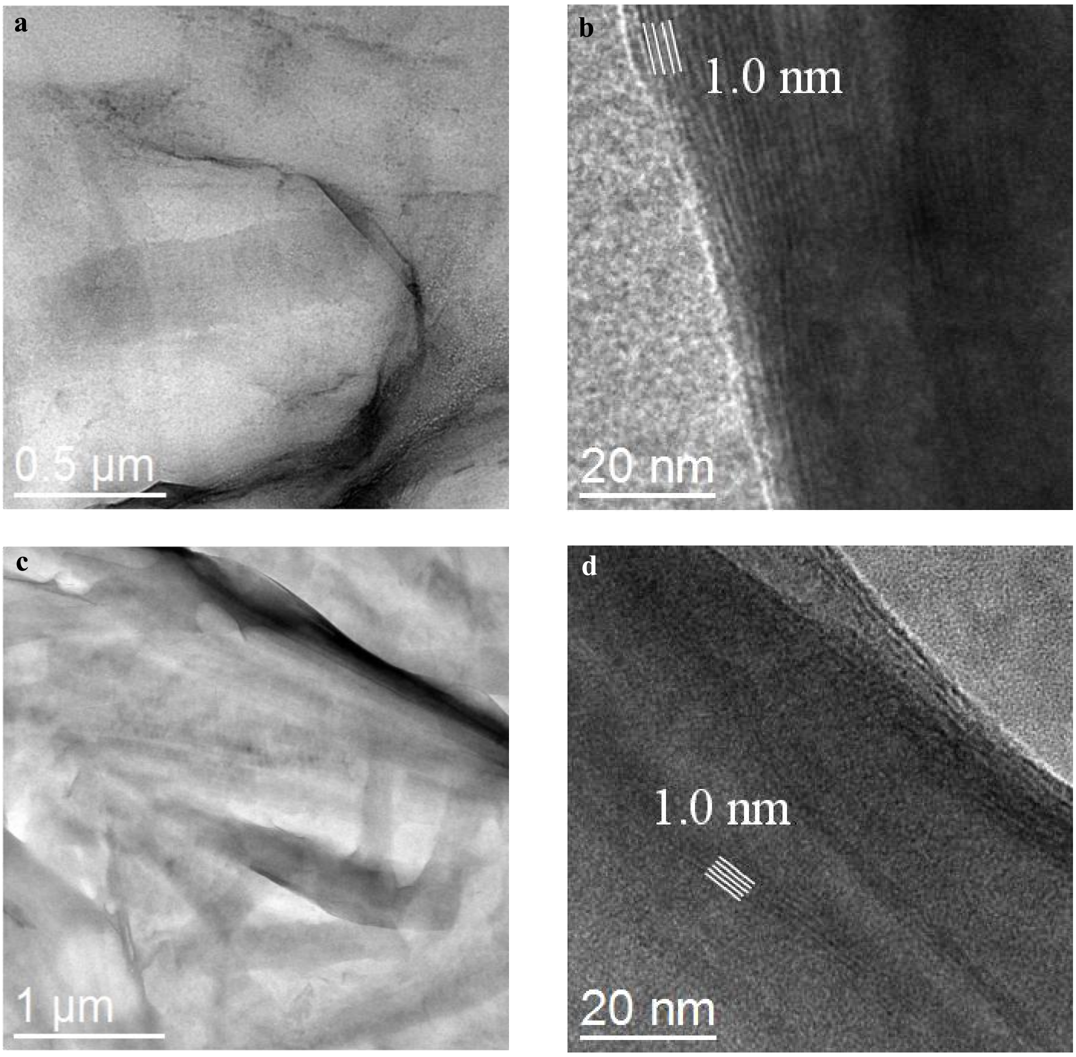

Fig. S9 TEM and HRTEM images of NAu-2 after 72 h of bioreduction. (a, b) Bioreduction of NAu-2 in the absence of ESHA, (c, d) Bioreduction of NAu-2 in the presence of ESHA.

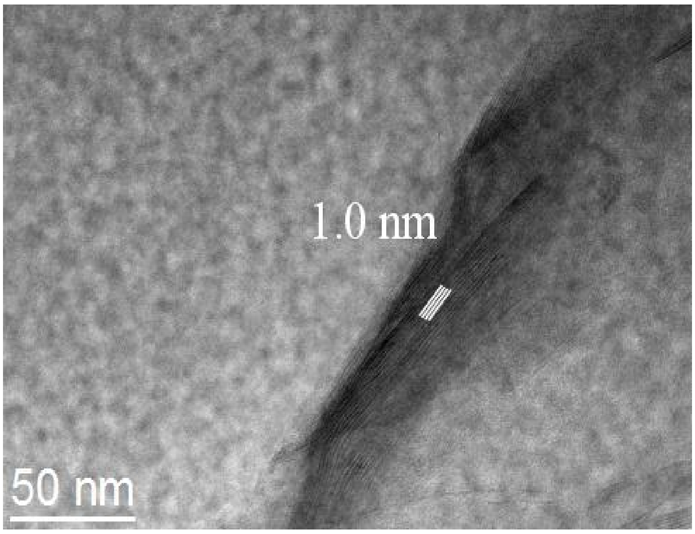

Fig. S10 HRTEM images of illite precipitate having 10 Å lattice fringes observed after NAu-2 bioreduction in the presence of ESHA.

Table S1 The polarity index[2] ((N+O)/C) of ESHA before and after adsorption. Conversions represent the mean ± deviation (n = 3).

Sample	C (%)	H (%)	N (%)	O[a] (%)	(N+O)/C[b]
ESHA	59.04 ± 3.11	3.26 ± 0.41	3.98 ± 0.15	33.70 ± 2.12	0.64± 0.00
Unbound HA	55.46 ± 5.13	1.85 ± 0.09	0.25 ± 0.01	42.43 ± 4.08	0.77 ± 0.00

[a] It was calculated by difference from 100 and sum of C, H and N.
[b] Atomic ratio of sum of nitrogen and oxygen to carbon.

Table S2 Integration results of the ^{13}C NMR spectra of the whole ESHA and the unbound ESHA after adsorption by NAu-2.

	distribution of C chemical shift (ppm), %								
Sample	0-60	60-96	96-108	108-145	145-162	162-220	aliphatic C[a], %	aromatic C[b], %	aliphaticity[c] (%)
Whole ESHA	30.11	12.92	4.09	33.70	6.23	12.95	47.12	39.93	54.13
Unbound ESHA	27.98	10.18	3.89	39.12	6.20	12.63	42.05	45.32	48.64

[a] Aliphatic C = total aliphatic carbon region (0-108 ppm).
[b] Aromatic C = total aromatic carbon region (108-162 ppm).
[c] Aliphaticity = aliphatic C (0-108 ppm)/sum of aliphatic C and aromatic C (0-162 ppm).

Table S3 The area integration of electron accepting capacity (EAC) and electron donating capacity (EDC) in electrochemical experiment. Conversions represent the mean ± deviation (n = 3).

Sample	EAC (e^-)	EDC (e^-)
NAu-2+unbound ESHA	1742.1 ± 8.1	503.3 ± 1.9
NAu-2y+whole ESHA	1374.7 ± 10.1	461.8 ± 4.0
NAu-2+sorbed ESHA	1220.7 ± 13.8	286.2 ± 0.7
NAu-2	313.5 ± 1.9	184.7 ± 0.2

Table S4 ICP-MS analysis of different cation concentrations in solution after bioreduction. Conversions represent the mean ± deviation (n = 3).

Element	Without HA (mg l^{-1})	With HA (mg l^{-1})
Al	U. D.[a]	3.603 ± 0.112
Si	5.113 ± 0.412	42.290 ± 0.110
K(+100 mg l^{-1})[b]	109.202 ± 3.124	90.100 ± 0.332
Mg	1.488 ± 0.176	1.813 ± 0.091
Ca	2.907 ± 0.112	4.061 ± 0.202

[a] Undetectable.

[b] There was 100 mg l^{-1} KCl in PIPES buffer solution (see Methods).

Table S5 Summary of studies to date which have quantified the extent of clay minerals NAu-2 bioreduction w/ and w/o electron shuttle.

Clay mineral concentration (g/l)	Bacterial strain	Electron transfer mediator	Fe(III) reduction extent (%) / Reaction time	Reference
1.0	*S. oneidensis* MR-1	/	22.0/168 h	This study
		10 mg/l AHA	25.2	
		50 mg/l AHA	28.1	
		100 mg/l AHA	32.1	
		200 mg/l AHA	39.5	
		10 mg/l SRHA	25.4	
		50 mg/l SRHA	28.9	
		100 mg/l SRHA	35.6	
		200 mg/l SRHA	44.8	
		10 mg/l ESHA	30.1	
		50 mg/l ESHA	36.4	
		100 mg/l ESHA	45.0	
		200 mg/l ESHA	49.9	
5.0		/	9.6	
		50 mg/l ESHA	21.0	
10.0		/	5.0	
		50 mg/l ESHA	20.1	
2.0	*S. putrefaciens* CN32 (CN32)	/	21.9/ 570 h	3
8.0	*S. algae* BrY	/	7.3/ 168 h	4
8.0		0.25 mM AQDS	26.3	4
8.0		1.0 mM Cystine	23.0	4
8.0		1.8 mM Cysteine	20.0	4

2.0	CN32	/	14.0-18.0/ 336 h	5
2.0		0.1 mM AQDS	28.0-32.0	5
5.0		/	15.7	6
5.0	*Methanothermobacter thermautotrophicus*	0.1 mM AQDS	26.9	7
5.0	*Thermoanaerobacter ethanolicus*	AQDS	40.4-43.7/ 600 h	8

References

1. Jason, D. R. & Michael, P. Proton-binding study of standard and reference fulvic acids, humic acids, and natural. *Geochim. Cosmochim. Ac.* **67**, 85–96 (2003).

2. Wang, K. & Xing, B. Structural and sorption characteristics of adsorbed humic acid on clay minerals. *J. Environ. Qual.* **34**, 342–349 (2005).

3. Luan, F., Liu, Y., Griffin, A. M., Gorski, C. A. & Burgos, W. D. Iron(III)-bearing clay minerals enhance bioreduction of nitrobenzene by Shewanella putrefaciens CN32. *Environ. Sci. Technol.* **49**, 1418–1426 (2015).

4. Liu, D., Dong, H. & Zhang, D. L. Smectite reduction by Shewanella species as facilitated by cystine and cysteine. *Geomicrobiol. J.* **31**, 53–63 (2014).

5. Jaisi, D. P., Dong, H. & Liu, C. Influence of biogenic Fe(II) on the extent of microbial reduction of Fe(III) in clay minerals nontronite, illite, and chlorite. *Geochim. Cosmochim. Ac.* **71**, 1145–1158 (2007).

6. Jaisi, D. P., Dong, H. & Liu, C. Kinetic analysis of microbial reduction of Fe(III) in nontronite. *Environ. Sci. Technol.* **41**, 2437–2444 (2007).

7. Zhang, J., Dong, H., Liu, D. & Agrawal, A. Microbial reduction of Fe(III) in smectite minerals by thermophilic methanogen Methanothermobacter thermautotrophicus. *Geochim. Cosmochim. Ac*. **106**, 203–215 (2013).

8. Zhang, G., Dong, H., Kim, J. & Eberl, D. D. Microbial reduction of structural Fe3+ in nontronite by a thermophilic bacterium and its role in promoting the smectite to illite reaction. *Am. Mineral.* **92**, 1411–1419 (2007).
